# Pulmonary Rehabilitation Using Modified Threshold Inspiratory Muscle Trainer (IMT) in Patients with Tetraplegia

**DOI:** 10.1155/2012/587901

**Published:** 2012-03-26

**Authors:** Funda Yasar, Canturk Tasci, Sema Savci, Ergun Tozkoparan, Omer Deniz, Arzu Balkan, Hayati Bilgic

**Affiliations:** ^1^Gulhane Medical School, Department of Pulmonary Medicine, Ankara, Turkey; ^2^Department of Pulmonary Rehabilitation, Hacettepe University, Ankara, Turkey

## Abstract

It is aimed to present the usefulness of inspiratory muscle trainer (IMT) in treatment of a 20-year-old male patient with diaphragmatic paralysis and tetraplegia due to spinal cord injury (SCI), and supporting effect of IMT in recovering from respiratory failure by rendering his diaphragm functions. The treatment was applied through the tracheostomy cannula by a modified IMT device. After applying IMT for three weeks, it was observed that the diaphragm recovered its functions in electromyography (EMG) test. As a result, in this study, we present a case where a patient could live without any respiratory device for the rest of his life with the help of modified IMT.

## 1. Introduction

The primary aim of the pulmonary rehabilitation is to increase the life quality and the comfort of the patients by helping them to adapt to daily activities as much as possible and to recover from respiratory failures. As it is wellknown, bilateral diaphragmatic paralysis is expected in patients with tetraplegia [1]. Respiratory failures are often inevitable in such patients. Invasive or non-invasive mechanical ventilation treatment is compulsorily needed by such patients to continue breathing [2]. As the diaphragm is the strongest inspiratory muscle, any dysfunction would cause severe respiratory failures. In spinal cord traumas, depending on the severity of the trauma, the diaphragmatic paralysis is frequent [3]. In diaphragmatic paralysis cases, drastic changes in lung volumes and decreases in inspiratory capacity and expiratory reserve volume are generally observed [4]. The most common methods of pulmonary rehabilitation in treatment of spinal cord traumas are using wheelchairs and arm-cranking exercise [5]. Inspiratory muscle trainer (IMT) device is generally applied to the patients with chronic obstructive pulmonary disease (COPD) [6]. This method helps to reduce the inspiratory workload by increasing the power of the inspiratory muscle and also to prevent the carbon dioxide retention. It is also frequently applied in cases with neuromuscular diseases such as Myasthenia Gravis and proved to be successful in reducing the need of mechanical ventilation [7]. Likewise, in this report, we present the successful application and the usefulness of modified IMT device through tracheostomy cannula on a patient with tetraplegia and diaphragmatic paralysis.

## 2. Case

**Figure 1:**
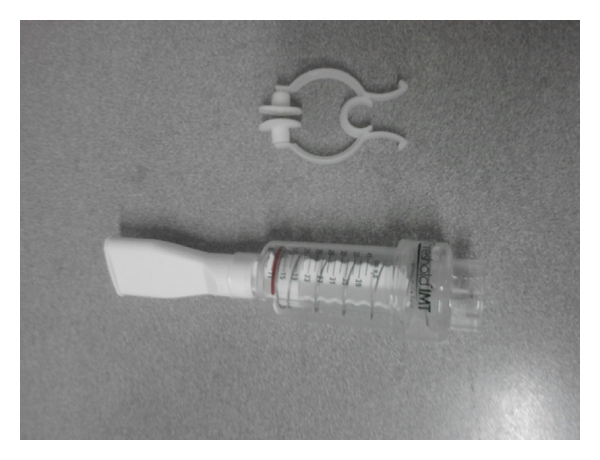
Threshold inspiratory muscle trainer.

**Figure 2:**
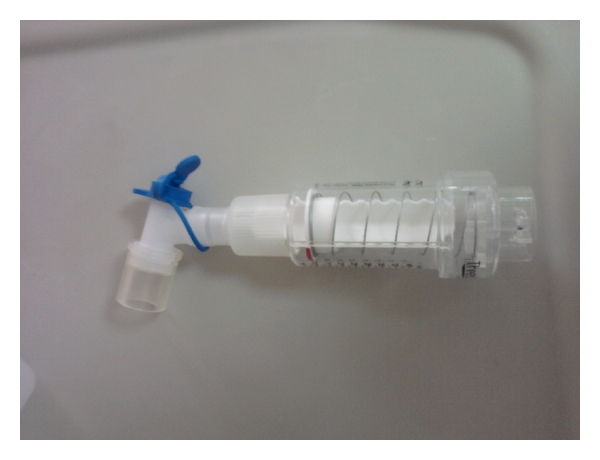
Modified threshold inspiratory muscle trainer.

**Figure 3:**
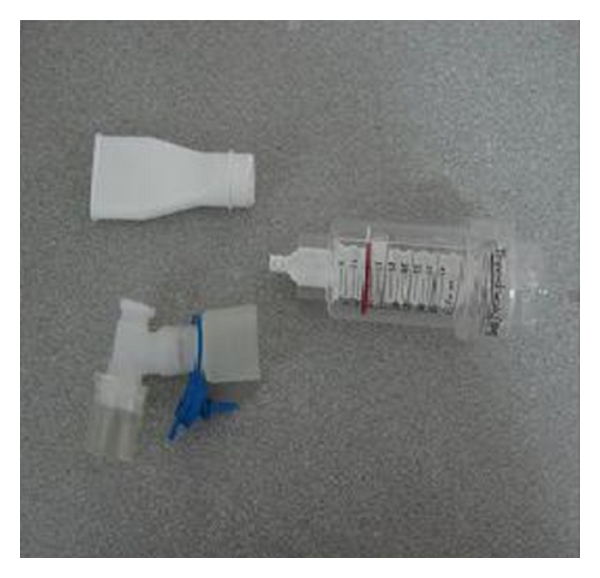
Threshold inspiratory muscle trainer.

**Figure 4:**
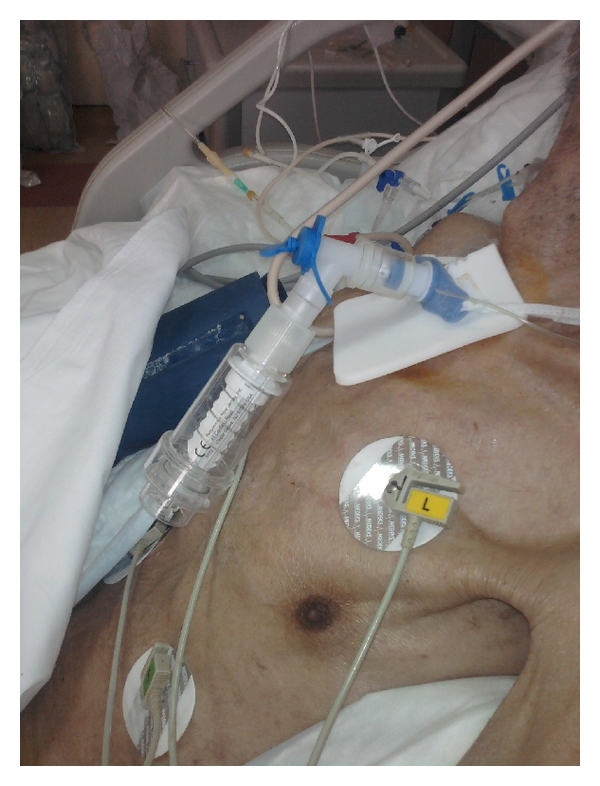
Implementation of the device on the patient.

Our case is a 20-year-old male patient with C5-6-7 vertebrae fracture because of falling from height. Surgical operation has been applied to him; however, he did not benefit from the surgery. Following the development of tetraplegia, respiratuary failure occurred in the patient, and invasive mechanical ventilator (IMV) was needed. While the patient was placed on IMV, the follow-up was conducted by trials both with controlled mode and the BIPAP mode; however, adequate tidal volume could not be reached due to the lack of diaphragmatic movement. Diaphragmatic paralysis was confirmed by electromyography (EMG). After the hospitalization of the patient, respiratory physiotherapy was utilized (respiratory exercises, postural drainage, etc.) regularly. This therapy has been applied for almost three months. During this period, adverse complications of IMV (ventilator associated pneumonia (VAP)) occurred, which had an undesirable effects on the physiotherapy due to insufficient respiratory reserve, weak cough reflexes, and so forth VAP is one of the major complications which increase morbidity in patients receiving mechanical ventilation. In the third month of the follow-up, the patient remained stable for the duration of mechanical ventilation on CPAP mode, and we estimated that pulmonary rehabilitation with Threshold Inspiratory Muscle Trainer would be useful for pulmonary functions of him (Figure 1). However, we had a difficulty in operating the IMT device through the tracheostomy cannula. Upon this problem, we modified IMT device as seen in Figures 2 and 3. For three weeks, this modified device has been applied to the patient through 20–30 breaths per day (Figure 4). The relative pressure was 12 cm H_2_O (20% of MIP) in first week, 16 cm H_2_O (30% of MIP) in second week, and 20 cm H_2_O (50% of MIP) in third week. At the end of the three weeks, the diaphragmatic movements of the patient have been observed and adequate tidal volume has been obtained during spontaneously breathing in room air. Sporadic IMT treatment has been applied, and, as a result, sufficient ventilation by mere room air has been achieved.

## 3. Discussion

Tetraplegia and diaphragmatic paralysis are often in severe spinal cord injuries. As a result of this, such patients have a great tendency to develop respiratory failures. They are bound to continue their lives through the help of respiratory supportive devices, but adequate respiratory volumes cannot be obtained through the use of invasive or noninvasive mechanical ventilators [4]. On the other hand, infectious complications of IMV, such as VAP, indicate the morbidity and the mortality in these patients [8]. Pulmonary rehabilitation is absolutely necessary in treatment procedures of these patients. There are numerous respiratory rehabilitation methods whose common target is to put the inspiratory and the expiratory muscle groups into effective use in breathing. In our case, the recovery in respiratory failure and diaphragm dysfunction was achieved.

A glance at the medical literature will help us to see that the major respiratory exercises are wheelchair and armcranking in patients with spinal cord injury [5]. In the medical reports, we see that the IMT is used on patients with COPD more often than other respiratory disorders [6]. Nevertheless, recently it has also been used on patients with neuromuscular diseases and respiratory failures due to spinal cord injuries. In the study of Petrovic et al., IMT has been applied to patients in addition to the application of NIV for a period of 4 months at nights, and there has been an increase in vital capacity for 9,6% [2]. Bailey et al. recorded and compared the maximum inspiratory pressure (MIP) values before and after the application of IMT for four weeks and observed statistically meaningful recovery in patients [9].

A contrastive analysis of pursed lip breathing and IMT has been conducted by Sutbeyaz et al. in patients with spinal cord trauma or stroke, and it has been observed that the exercise capacities and life qualities have been increased in the patients who received IMT. At the end of the study the lung volume of the patients who received IMT had a statistically meaningful recovery in comparison with those of patients who were treated with pursed lip breathing [10].

Liaw and colleagues investigated the efficacy and feasibility of home-based IMT in 26 patients with bronchiectasis in their randomized trial that have reached success by working 30 minutes per day for eight weeks [11]. They performed measurements including spirometry for evaluating the effects of treatment and observed an improvement both in inspiratory and expiratory muscle strength, but no effect on respiratory function and quality of life in patients with bronchiectasis. As seen in Liaw et al.'s study, pulmonary function test result is an objective criteria for evaluating the improvement in respiratory functions in such patients, but we could not be able to perform spirometry to our patient because of his physical condition. On the other hand we demonstrated the diaphragma movement with EMG which was absent before the IMT treatment and also a well recovary in respiratory function of the patient.

IMT diaphragmatic paralysis is present prior to application, and the application is running after the diaphragm was detected by Müller and colleagues [12] noted in the first year after trauma, such as improved respiratory muscle function shown without IMT. However, we think that our case is the contribution of IMT respiratory functions.

As a result, it has been deduced that, in cases with tracheostomy and chronic respiratory failure where respiratory rehabilitation treatments do not produce desired results, the diaphragm, being the biggest inspiratory muscle, can be put into use with the modified IMT application, and ideal results can be attained. It has been evaluated that the incremental application of this method in other cases, thus attaining more objective findings, will be more than appropriate.

## References

[1] Celli BR (2002). Respiratory management of diaphragm paralysis. *Seminars in Respiratory and Critical Care Medicine*.

[2] Petrovic M, Lahrmann H, Pohl W, Wanke T (2009). Idiopathic diaphragmatic paralysis-Satisfactory improvement of inspiratory muscle function by inspiratory muscle training. *Respiratory Physiology and Neurobiology*.

[3] Baydur A, Adkins RH, Milic-Emili J (2001). Lung mechanics in individuals with spinal cord injury: effects of injury level and posture. *Journal of Applied Physiology*.

[4] Sheel AW, Reid WD, Townson AF, Ayas NT, Konnyu KJ (2008). Effects of exercise training and inspiratory muscle training in spinal cord injury: a systematic review. *Journal of Spinal Cord Medicine*.

[5] Vrabas IS, Dodd SL, Powers SK (1999). Endurance training reduces the rate of diaphragm fatigue in vitro. *Medicine and Science in Sports and Exercise*.

[6] Geddes EL, Reid WD, Crowe J, O’Brien K, Brooks D (2005). Inspiratory muscle training in adults with chronic obstructive pulmonary disease: a systematic review. *Respiratory Medicine*.

[7] Fregonezi GA, Resqueti VR, Güell R, Pradas J, Casan P (2005). Effects of 8-week, interval-based inspiratory muscle training and breathing retraining in patients with generalized myasthenia gravis. *Chest*.

[8] DeVivo MJ, Black KJ, Stover SL (1993). Causes of death during the first 12 years after spinal cord injury. *Archives of Physical Medicine and Rehabilitation*.

[9] Bailey SJ, Romer LM, Kelly J, Wilkerson DP, DiMenna FJ, Jones AM (2010). Inspiratory muscle training enhances pulmonary O2 uptake kinetics and high-intensity exercise tolerance in humans. *Journal of Applied Physiology*.

[10] Sutbeyaz ST, Koseoglu F, Inan L, Coskun O (2010). Respiratory muscle training improves cardiopulmonary function and exercise tolerance in subjects with subacute stroke: a randomized controlled trial. *Clinical Rehabilitation*.

[11] Liaw M-Y, Wang Y-H, Tsai Y-C (2011). Inspiratory muscle training in bronchiectasis patients: a prospective randomized controlled study. *Clinical Rehabilitation*.

[12] Müller R, Peter C, Cieza A, Geyh S (2012). The role of social support and social skills in people with spinal cord injury-a systematic review of the literature. *Spinal Cord*.

